# A Focus on the Role of Dietary Treatment in the Prevention of Retinal Dysfunction in Patients with Long-Chain 3-Hydroxyacyl-CoA Dehydrogenase Deficiency: A Systematic Review

**DOI:** 10.3390/children12030374

**Published:** 2025-03-17

**Authors:** Evelina Maines, Giorgia Gugelmo, Nicola Vitturi, Alice Dianin, Laura Rubert, Giovanni Piccoli, Massimo Soffiati, Vittoria Cauvin, Roberto Franceschi

**Affiliations:** 1 Division of Pediatrics, Santa Chiara General Hospital, APSS Trento, 38122 Trento, Italy; massimo.soffiati@apss.tn.it (M.S.); vittoria.cauvin@apss.tn.it (V.C.); roberto.franceschi@apss.tn.it (R.F.); 2 Division of Metabolic Diseases, Department of Medicine, Padova University Hospital, 35128 Padova, Italy; giorgia.gugelmo@unipd.it (G.G.); nicola.vitturi@aopd.veneto.it (N.V.); 3 Inherited Metabolic Diseases Unit and Regional Centre for Newborn Screening, Diagnosis and Treatment of Inherited Metabolic Diseases and Congenital Endocrine Diseases, University Hospital of Verona, 37126 Verona, Italy; alice.dianin@aovr.veneto.it (A.D.); laura.rubert@aovr.veneto.it (L.R.); 4 CIBIO—Department of Cellular, Computational and Integrative Biology, University of Trento, 38123 Trento, Italy; giovanni.piccoli@unitn.it

**Keywords:** diet, retinopathy, LCHADD

## Abstract

**Background**: Long-chain 3-hydroxyacyl-CoA dehydrogenase deficiency (LCHADD) is an inborn error affecting fatty acid β-oxidation (FAO). Differently than other FAO deficiencies, LCHADD patients may develop progressive retinopathy and peripheral neuropathy. The pathogenesis of retinopathy is not completely understood, and the role of dietary interventions in preventing the development of retinopathy remains uncertain. We examined the literature to assess the impact of the dietary management of LCHADD patients on retinopathy prevention. **Methods**: Our systematic search included studies published in the last 20 years according to PRISMA guidelines. The aims of the review were to analyze the correlation between retinopathy and the following: (1) age at first metabolic decompensation and/or at the start of the dietary treatment, (2) chronic dietary treatment, (3) emergency regimens, (4) other nutritional supplements. The protocol was registered in PROSPERO, and evidence was assessed using the GRADE system. **Results**: Seven full papers were identified according to search criteria, with only four including meaningful data. Early presentation of the disease, acute neonatal symptoms, and a suboptimal chronic treatment control were associated with more aggressive retinopathy and a poorer sight outcome. The number of metabolic decompensations and/or hospitalizations were also positively correlated with vision loss. Chronic fat modulation in the diet had less impact than emergency treatments. The role of other nutritional supplements was not well defined. **Conclusions**: Newborn screening may improve retinal outcomes. Nevertheless, early treatment adopting the current LCHADD therapeutic regimen can often only delay the onset of retinopathy. Clearly, our current treatment strategies are not adequate and retina-specific treatments are needed. The optimal composition of the diet, the role of fasting limitation, and the benefits of some nutritional supplements deserve further investigations.

## 1. Introduction

Long-chain 3-hydroxyacyl-CoA dehydrogenase deficiency (LCHADD) (OMIM #609016) is an autosomal recessive inborn error affecting the metabolism of long-chain fatty acids. The enzyme long-chain 3-hydroxyacyl-CoA dehydrogenase (LCHAD) is part of the mitochondrial trifunctional protein (TFP) and catalyzes the third (out of four) step along mitochondrial fatty acid β-oxidation (FAO). TFP encompasses three enzymatic activities: long-chain enoyl-CoA hydratase, long-chain 3-hydroxyacyl-CoA dehydrogenase, and long-chain 3-ketoacyl-CoA thiolase. In individuals with LCHADD, only LCHAD activity is missing. TFP deficiency (TFPD) is instead characterized by the loss of the activity of all three enzymes [1].

Clinical symptoms mainly develop during fasting, febrile infections, and gastroenteritis and affect organs needing long-chain fatty acids (LCFA) as a primary energy source, such as the heart and skeletal muscles. The typical features of metabolic decompensation are hypoketotic hypoglycemia, rhabdomyolysis, cardiomyopathy, and hepatic dysfunction [1,2]. Beyond acute symptoms, LCHADD is associated with well-characterized long-term complications, such as retinopathy and peripheral neuropathy. For these reasons, many countries now include LCHADD in their screening programs for newborn infants [1].

The incidence of LCHADD based on newborn screening (NBS) data from Australia, Germany, and the United States was estimated at 1:250,000 [1]; it is more frequent in the Finnish population, where it reaches 1: 62,000 [2].

Chronic dietary management is based on fasting limitation and a high-carbohydrate, low-fat diet with the limited intake of exogenous fat (25–30% of total calorie intake), mainly medium-chain triglycerides (MCT, 20–25% of total calories intakes) and long-chain triglycerides (LCT, 5–10%) [3,4,5]. Triheptanoin or heptanoate (odd-chain medium length fatty acids, C7) may provide a fatty acid substrate that can be used for energy [1]. Since children with LCHADD have fasting intolerance, night feeds could be necessary. Emergency treatment with glucose polymers or glucose infusion is a standard treatment for preventing catabolic episodes during illness [3,4,5].

Differently from other FAO deficiencies, patients with LCHADD and TFPD may develop a progressive pigmentary retinopathy and peripheral neuropathy [1,6]. Retinopathy is usually more common in LCHADD than in TFPD [7]. It may evolve to chorioretinal atrophy of the central fundus, high myopia, posterior staphyloma, and vision impairment [8]. Four different stages for retinopathy in LCHADD have been described [9]. Visual impairment is present from stage 3 onward [9]. Recently, a novel staging system of LCHADD retinopathy was developed. The four previous delineated stages were maintained but substages A and B in stages 2 to 3 were created to achieve a more detailed differentiation [10].

The pathogenesis of retinopathy in LCHADD is not completely understood [8]. Factors, such as the overall impact of toxic metabolites, namely 3-hydroxyacylcarnitines (3-OHACs) or relative fatty acids, energy deficiency, number of clinical decompensations, or episodes with hypoglycemia, all may trigger the pathological process [8]. In addition, children with LCHADD are treated with a low-fat diet, thus with a limited intake of docosahexaenoic acid (DHA) [11], crucial for visual and brain development and immune functions [12,13].

Dietary interventions are effective in sustaining patient survival; however, their impact on the development of retinopathy remains uncertain. Therefore, we searched the literature to assess the influence of dietary management on retinopathy in LCHADD patients.

The deterioration of vision is a severe consequence of LCHADD, and the specific pathophysiology is still not known. The aim of the review is to examine the impact of dietary interventions in the prevention of retinal dysfunction in patients with LCHADD. 

## 2. Materials and Methods

### 2.1. Protocol and Registration

This systematic review was developed according to the preferred reporting items for systematic reviews and meta-analyses (PRISMA) guidelines [14]. The protocol was registered in International Prospective Register of Systematic Reviews (PROSPERO), registration number [CRD42025633361] (https://www.crd.york.ac.uk/prospero/ accessed on 3 January 2025).

### 2.2. Search Strategy

We searched the relevant electronic databases (Pubmed, The Cochrane Library, Clinicaltrial.gov, International Clinical Trials Registry Platform) for studies published between 1 January 2004 and 1 September 2024. Search terms or “MESHs” (MEdical Subject Headings) for this systematic review included different combinations: “LCHAD” or “LCHADD” or “long-chain 3-hydroxyacyl-CoA dehydrogenase deficiency” AND “retinopathy”.

To avoid missing any relevant studies, we also screened the reference list derived from the eligible studies.

### 2.3. Criteria for Study Selection

We conducted a systematic search of the literature according to the PICOS model (Population, Intervention, Comparison, Outcomes and Study design):

Population: humans with LCHADD

Intervention: early dietary intervention (NBS)

Comparison: late or no dietary treatment (not through NBS), specific nutritional supplementations

Outcomes: the association of retinopathy with the following: (1) the role of age at first metabolic decompensation and/or age at starting dietary treatment, (2) the role of chronic dietary treatment, (3) the role of emergency regimens, (4) the role of other nutritional supplements.

Study design: randomized clinical trials (RCTs), observational studies (cohort, case-control, cross-sectional studies), exploratory studies, mix of qualitative and quantitative studies, case series.

Inclusion criteria were as follows: (i) study population, humans with LCHADD; (ii) study type, RCTs, observational studies (cohort, case series, cross-sectional studies), exploratory studies, mix of qualitative and quantitative studies; (iii) data on retinopathy, its relationship with diet, age, and genotype; (iv) publication date, last 20 years (2004–2024). Only full papers were included, whereas abstracts only were not included.

Exclusion criteria were as follows: (i) animal data; (ii) full paper not available; (iii) review, single case report. A language other than English was not an absolute exclusion criterion. 

### 2.4. Data Extraction and Management

Two independent investigators (EM, GG) screened the title and abstract of all the studies identified using the search strategy. Any discrepancies between them were resolved by consensus or through consultation with a third investigator (AD). After abstract selection, two investigators conducted the full paper analysis (EM, GG).

The following characteristics were evaluated for each study: (i) reference details, authorship(s); published; year of publication; period in which the study was conducted; other relevant cited papers; (ii) study characteristics, study design, topic, treatment period, follow-up duration, country; (iii) population characteristics, number of participants, age, diet, genotype; (iv) methodology, assessment of dietary management; (v) main results, effect of dietary management on retinopathy in LCHADD patients, relation with age at diagnosis, diet, genotype, and other factors.

### 2.5. Assessment of the Certainty of the Evidence

We used the GRADE approach (Grading of Recommendations Assessment, Development and Evaluation) to rank the quality of evidence (www.gradeworkinggroup.org accessed on 2 September 2024) for the included studies [15]. Two authors (EM, GG) independently assessed the certainty of the evidence for each of the outcomes. The GRADE approach results in an assessment of the certainty of a body of evidence and of the allocation to one of four grades:

High: further research is very unlikely to change confidence in the estimate of the effect;

Moderate: further research is likely to have an important impact on confidence in the estimate of the effect and may change the estimate;

Low: further research is very likely to have an important impact;

Very low: any estimate of effect is very uncertain.

In the case of a possible bias in the study design, imprecision of estimates, inconsistency across studies, indirectness of the evidence, and publication bias, we decreased the level of certainty by one or two levels as suggested by the GRADE guidelines [15].

## 3. Results

**Figure 1:**
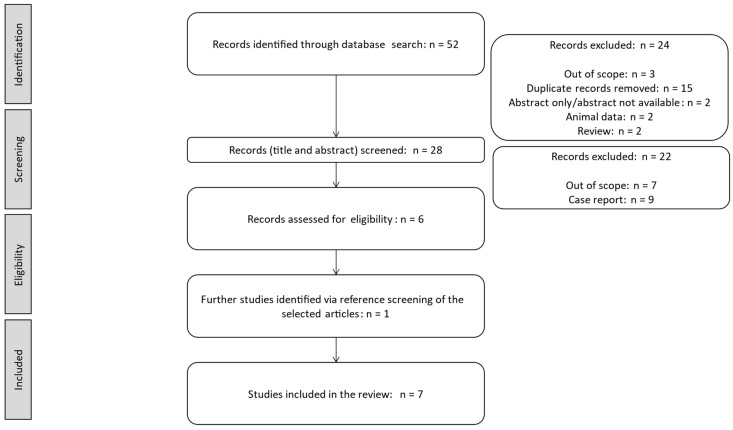
PRISMA flow chart for the identification, screening, eligibility, and inclusion of the selected studies.

Seven full papers were identified according to the search criteria (Figure 1, PRISMA flow chart). 

The selected studies are listed and described in Table 1.

The studies included more than 120 LCHADD patients from various countries, such as Sweden, Finland, Austria, Germany, and the USA. Four studies included only pediatric patients [6,16,17,18], while three studies included both children and adults [8,19,20]. Some patients are included in more than one study [8,18].

In four studies, the majority of patients were diagnosed through newborn screening (NBS) or based on a family history [16,19,20]. In others, the patient cohort was mainly represented by patients diagnosed later following the onset of symptoms, with varying diagnostic approaches across the cohorts [6,8,18]. In the study of Gillingham et al. [17], the timing of diagnosis was not reported. 

Specifically, 31 patients were diagnosed through NBS [8,16,19], while 8 patients were diagnosed based on a family history [19]. A group of 84 patients was diagnosed after presenting with symptoms [6,8,16,18,19,20].

Most participants bore the 1528G>C mutation [6,16,17,18,19,20], which is the most common pathogenic variant in LCHADD [1], though a few studies also included patients with other genetic variants [17,18,19,20]. The follow-up periods ranged from a few months to several years. Patients from different regions were studied with different follow-up protocols.

Concerning the main dietary strategies, all studies reported that the LCHADD patients were treated with a low-fat high-carbohydrate diet [6,8,16,17,18,19,20]; in most studies, MCT supplements were started at the time of diagnosis [6,16,17,18,19]. In some studies, the reported LCFA intake of patients ranged from 3 to 24% of calorie intake [6,16,17,18]. All authors also reported the administration of essential fatty acids or DHA [6,8,16,17,18,19,20] or soluble vitamins [6].

Continuous overnight feeding was experimented in some studies [6,8]. Some patients were treated with triheptanoin [16,19]. Emergency regimens provided glucose polymer supplementation and IV glucose infusion [6,8,16,18]. 

Among the selected studies, four included both ocular and metabolic assessments as part of their follow-up, allowing for a comprehensive evaluation of both outcomes [6,8,18,19]. Two studies focused primarily on ocular assessments, such as visual acuity, fundus imaging, and optical coherence tomography (OCT) [17,20]. Additionally, one study emphasized metabolic follow-up with a focus on fatty acid profiles and acylcarnitines but did not provide detailed ocular evaluations [16].

The assessment of visual function varied across studies but commonly included measures of visual acuity, such as the best corrected visual acuity (BCVA) [8,18,20], electronic visual acuity tester with logarithmic minimal angle of resolution (LOGMAR) [19], or visual evoked potential (VEP) test [17]. Fundus imaging was evaluated using OCT [8,18,20], fundus autofluorescence, or infrared imaging [20]. Electrophysiological tests, including ERG, were reported in multiple studies [8,17,18,19]. Additional evaluations encompassed stereopsis, color vision, ocular alignment, slit-lamp examinations, ophthalmoscopy, and retinoscopy [8,18], as well as contrast sensitivity and visual field testing [19].

In the selected studies, four were retrospective [8,16,18,20], two were prospective [17,19], and one combined both retrospective and prospective elements [6].

### 3.1. Outcomes

#### 3.1.1. Prevalence of Retinopathy

Fahnehjelm et al. [8] reported that more than 80% of patients (age 3–26 years) developed pathological or subnormal retinal function documented via electroretinography (ERG). Immonen et al. [6] also reported retinopathy in most (81%) of the LCHADD patients (age 1–11 years) who survived more than 6 months after the diagnosis. These data are confirmed by the study of Karall et al. [16], where 8 out of 14 patients (age 0.9–15.3 years, median 7.6 years) developed retinopathy at a mean age of 47.6 months, and by the study of Dulz et al. [20], where all patients (mean age 14.9 years, range 7.9–23.9 years) showed retinal alterations. Gillingham et al. [19] also reported a very high incidence of retinopathy in their cohort of patients (up to 92% of patients diagnosed symptomatically, age 7–31 years).

#### 3.1.2. The Role of Age at First Metabolic Decompensation and/or Age at Starting Dietary Treatment

Gillingham et al. [19] compared the visual outcomes between 12 patients with LCHADD or TFPD diagnosed symptomatically and 28 patients who were diagnosed via NBS or by judging the family history. The most relevant difference in dietary treatment between the groups was the age at the start (in the newborn period for patients diagnosed by NBS/family history and between 7 days and 3 years of life for the others). Both age and mode of presentation (symptomatic vs. detected through NBS) were found to be significant yet independent factors associated with visual outcomes. Visual acuity, rod-and cone-driven amplitudes on ERG, contrast sensitivity scores, and visual fields were all significantly worse among participants diagnosed symptomatically compared to via NBS (92% vs. 68%).

The role of age and symptoms at the start of the dietary treatment is also reported in other studies.

Karall et al. [16] reported the mean age of the retinopathy diagnosis across different patient groups based on how they were diagnosed with LCHADD. Among patients born before LCHADD was included in the NBS program—when diagnosis was primarily based on the clinical presentation—the mean age of retinopathy diagnosis was 51.6 months, with LCHADD diagnosed at a mean age of 10.3 months (range: 3–23 months). In patients born after the introduction of NBS but who developed symptoms before their NBS results were available, retinopathy was diagnosed at a mean age of 39 months, with LCHADD diagnosed at a mean age of 15 days. In contrast, asymptomatic patients identified through NBS were diagnosed with retinopathy at a mean age of 53 months. 

Fahnehjelm et al. [8] examined 12 Swedish LCHADD patients (age 3–26 years), with only 2 diagnosed via NBS. All started the dietary treatment at the time of diagnosis (range 1 week-13 months, mean 5.5 months). ERG was performed on 11 patients (median age at first ERG: 6 years, 0.7–16.5 years; median age at the latest ERG: 14 years, range 3–22 years). It was pathological in 5 patients and subnormal in 4 patients. Four of the five patients with pathological ERG findings had severe symptoms at birth, including neonatal hypoglycemia, and the fifth patient was symptomatic at birth. The four patients who did not have hypoglycemic events in the newborn period had normal or borderline ERG findings. 

Dulz et al. [20] reported six LCHADD cases, three diagnosed via NBS (age at follow-up 8-14,8 years), one diagnosed within the first year of life (age at follow-up 14.8 years) and two diagnosed later with symptoms (age at follow-up 23.5–24 years). All patients showed retinal alterations, but early diagnosis and treatment were associated with a better clinical outcome and a longer preservation of visual function. Among symptomatic patients, only one showed mild retinal involvement at the time of diagnosis.

Fahnehjelm et al. [18] also reported that retinopathy was less pronounced in patients with an early diagnosis.

These findings suggest that preventing metabolic decompensation in proximity to birth may prevent the retinal damage in LCHADD. 

Nevertheless, some studies described that visual outcomes were improved upon the early diagnosis allowed by NBS, but visual function was impaired along with aging [8,19,20].

#### 3.1.3. The Role of Chronic Dietary Treatment and Metabolic Control

Gillingham et al. [17] reported that when patients with LCHADD are generally healthy, eat regular meals, and consume a low-fat diet, 3-OHAC levels fall and the progression of the retinopathy is slowed. The authors measured plasma levels of 3-OHACs once per year in 14 LCHADD/TFPD patients followed over 5 years. Notwithstanding the limits of such 3-OHAC and hydroxy fatty acid analyses during the follow-up period, they observed a strong negative correlation between the 3-OHAC concentration and phototransduction, as measured via ERG (*p* = 0.0038). In addition, patients with sustained low plasma 3-OHACs maintained higher ERG amplitudes compared to patients with chronically high 3-OHACs. The authors concluded that lowering 3-OHAC by-products slows the progression of retinopathy. Moreover, they observed that the LCHADD/TFPD genotype correlates with 3-OHACs levels and retinopathy progression.

In the study of Fahnehjelm et al. [8], the chronic metabolic control was determined by measuring the mean values of dicarboxylic acids and/or 3OHAC levels from the diagnosis (2 patients diagnosed via NBS and 10 at a median age of 5.5 months, range 1 week-13 months) to the first ERG examination (median age at first: 6 years, range 0.7–16.5 years). Thereafter, the authors measured the mean values of dicarboxylic acids and/or acylcarnitines between subsequent ERG examinations (median age at the latest ERG: 14 years, range 3–22 years). Mean values below the upper reference limits were given a score of one and considered good treatment control; mean values slightly above the upper reference limits were given a score of two and considered acceptable treatment control; mean values in excess of the upper reference values by more than two standard deviations were considered poor treatment control and given a score of three. The authors did not find any correlation between the ERG amplitude or latency and 3-OHACs levels in three patients who presented with ERG deterioration over age. Nevertheless, three of five patients with pathological ERG findings scored three, indicating a poor treatment control. The other two patients with pathological ERG scored two, which indicated acceptable treatment control. All six patients with normal or subnormal ERG findings had acceptable or good treatment control, with a score of one or two, except for a case who had poorer treatment control. The calculated composite score accounted for all the risk factors, namely symptoms at diagnosis, treatment control, and later clinical symptoms. Combined scores had a good correlation with ERG and latencies. 

Previously, Fahnehjeim et al. [18] developed a clinical scoring system evaluating maternal, prenatal, neonatal, and somatic risk factors for long-term ocular complications, with scores (one to three points for each score, with higher values indicating worse factors) correlated with ocular findings and ERG results. Notably, six children had a total clinical score of 20 or more, suggesting a high risk for ocular complications; all but one of these high-scoring children displayed severe chorioretinopathy (grade 3 or 4). Longitudinal ERG assessments revealed a progressive retinal dysfunction in the majority (7 of 10) of children, with notable retinal changes often preceding measurable declines in visual function.

The recent prospective study by Gillingham et al. [19] demonstrated that while current dietary interventions reduce toxic intermediates (e.g., 3-OHFAs) associated with retinal degeneration, they do not fully normalize them. Additionally, older participants exhibited the most severe retinal changes suggesting a potential cumulative effect of toxic metabolites.

On the contrary, Dulz et al. [20] observed no obvious correlation between 3-hydroxy fatty acid (3-OHFA) levels and the stage of ocular progression. All the patients were treated with a low-fat diet, but strict adherence to the therapy was only observed in one patient. 

#### 3.1.4. The Role of Emergency Regimens

Some results suggest that the number of metabolic decompensations and/or hospitalizations experienced by the LCHADD patients correlates with the vision loss. Fahnehjelm et al. [8] reported that the incidence of pathological or subnormal retinal function is reduced in patients with strict dietary treatment and good metabolic control. 

During the metabolic crises, the levels of 3-OHACs and other by-products are likely high. Gillingham et al. [17] observed that patients that carry at least one 1528G>C allele, follow the recommended diet, and have fewer metabolic decompensations are characterized by lower 3-OHACs and slower progression of the retinopathy. Decreasing the number of metabolic crisis delays or slows the progression of retinopathy, suggesting that these moments of stress accelerate the progression of retinopathy. 

On the contrary, in another study [20], no obvious correlation linked 3-OHFA levels, the number of metabolic crisis, and the stage of ocular progression. 

#### 3.1.5. The Role of Other Nutritional Supplements

Gillingham et al. [17] observed that over 50% of the LCHADD/TFPD patients had plasma DHA levels below the normal range. Thus, DHA supplementation, particularly in DHA-deficient patients, has been postulated to prevent or delay the progression of retinopathy. The authors observed that visual acuity, as determined based on the visual evoked potential test (VEP), appeared to increase upon DHA supplementation (65–130 mg/day) (*p* = 0.051) and correlates with plasmatic DHA concentrations (*p* = 0.075, R2 = 0.31). Even some of the patients with normal plasmatic DHA levels at baseline experienced a visual acuity improvement upon DHA supplementation. 

Nevertheless, in other studies, most patients who developed retinopathy received DHA supplementation from an early age at a dosage in the range of the recommended intake for healthy individuals [6,8,16]. Despite DHA supplementation, visual function impaired along with aging in some cases [8]. 

Fahnehjelm et al. [18] also reported that one patient demonstrated a decreased rod and cone amplitude during a 2-year period despite supplementation with high levels of DHA. 

Immonen et al. [6] reported low plasma vitamin A concentrations in some patients before the diagnosis of LCHADD and even after the initiation of the diet. In this study, retinopathy was observed in 9/11 patients who survived more than 6 months after the diagnosis. The authors did not report any correlation between vitamin A levels and the risk of retinopathy. Despite the known role of vitamin A in the visual function, no studies have specifically investigated the potential role of vitamin A deficiency in developing retinopathy in LCHADD patients.

Supplementation with MCT ranging from 20 to 80% of fat intake [6,16,19] or C7 (triheptanoin or heptanoate) [16,19] has been tested. MCT may mitigate toxic intermediate build-ups and support metabolic stability; nevertheless, despite supplementation, visual and retinal deterioration continues, and older participants exhibited the most severe retinal changes [19].

Regarding C7 supplementation, a case series of 14 patients revealed no significant difference in clinical outcomes between those receiving supplementation and those who did not [16]. Patient stabilization over time was attributed to the anaplerotic effect of C7, although it may have simply been due to age-related factors, such as fewer infections with an increasing age.

## 4. Discussion

Retinopathy is encountered in a significant subset of LCHADD and TFPD patients and has a substantial impact on their quality of life due to the visual impairment. The role of clinical contributors, such as diet, in the pathogenesis of this severe long-term complication is still not completely known. A better understanding of the role of dietary interventions is important for improving the management of the patients. 

The retina has traditionally been thought to be a glycolytic tissue, meaning oxidizing glucose to gather energy rather than relying on the β-oxidation of fatty acids. However, the expression of β-oxidation proteins, such as TFP in the retina [9], and the progressive retinopathy associated with TFP genetic disorders suggest that FAO has a role in retinal metabolism [7]. Recent studies have confirmed that the retinal pigment epithelial (RPE) cells require FAO for maintaining the metabolic homeostasis of the retina [21]. 

LCHADD mouse models oxidize less fat and more glucose than wild-type mice, suggesting a potential role of metabolic changes in the pathogenesis of the retinopathy [22]. 

Nevertheless, in most genetic disorders where FAO is disrupted, patients do not present vision loss suggesting that impaired FAO is not sufficient to cause retinal degeneration. Clearly, the pathogenetic factors underlying visual impairment in LCHAD and TFP patients must still be discovered.

The pathophysiology of retinopathy in LCHADD has yet to be fully elucidated, and several theories have been proposed. Recent data prompted the hypothesis of a complex multifactorial pathogenesis. In particular, DeVine et al. [23] observed that induced pluripotent stem cells (iPSC)-derived mature RPE cells carrying the homozygous c.1528G>C common pathogenic variant for LCHADD oxidize less fat, produce fewer ketones, accumulate 3-OHACs, and have reduced tolerance to oxidative stress and lipid peroxidation. Tucci et al. [24] described, in the fibroblasts of LCHADD patients, an altered composition of cardiolipins, sphingomyelin, and ceramides, suggesting the involvement of complex lipids in the neurodegenerative process. Nevertheless, these data have been retrieved from fibroblasts or iPSC-derived cells and may not allow for a full extrapolation to patients. 

Thus, it is still relevant to investigate the role of dietary interventions in clinical contexts.

The first outcome of this systematic review was identifying the relevance of age at first metabolic decompensation and/or age at starting dietary treatment.

Some authors reported that retinopathy was less pronounced in patients with an early diagnosis [8,16,18,19,20].

In particular, Gillingham et al. [19] reported that there were significant differences in retinal images, visual acuity, contrast sensitivity, visual fields, and retinal function between participants diagnosed via NBS/family history and those who presented symptomatically and were diagnosed later in childhood. Deterioration in the ERG findings seemed to progress more rapidly in patients with dramatic neonatal symptoms, such as neonatal hypoglycemia.

These findings suggest that preventing metabolic decompensation in proximity to birth may be an important protective factor in the development of retinal damage in LCHADD. Nevertheless, in some studies, visual outcomes were improved through NBS, but there was still a visual impairment along with aging [8,19,20]. Thus, while the early initiation of dietary treatment appeared to mitigate the severity of the retinopathy, it did not fully prevent retinal changes. 

Severe energy deficiency at birth could cause the deterioration of vision. Indeed, the ß-oxidation process may be particularly important in energy-demanding organs, including the retina [9]. Patients diagnosed via NBS are less frequently affected by episodes of hypoglycemia, hypotonia/myopathy, hepatopathy, and cardiomyopathy as presumably a sufficient energy supply, the reduction of long-chain acyl CoA esters, and other toxic metabolites follow the diagnosis [1,2].

Alternatively, the lower total accumulation of toxic metabolites in RPE cells in patients identified with NBS may assure prolonged cellular survival under optimized dietary intake. In the case of a late diagnosis, a massive accumulation of toxic metabolites might lead not only to irreversible damage of some RPE cells prior to the diagnosis but also to the significant accumulation of toxic species in the functionally intact RPE cells [25]. Additional oxidative stress and inflammatory reactions might further precipitate the degeneration of adjacent cellular cells as seen in age-related macular degeneration [26]. Some studies highlighted that the age at symptom presentation and starting dietary treatment may also be critical beyond the neonatal period. In some patients, the mean age at which retinopathy was first diagnosed has been associated with the mean age of the LCHADD diagnosis [16,19].

The second outcome was the role of chronic dietary treatment. 

In some studies, the risk of retinal dysfunction seems to be ameliorated by good adherence to chronic dietary treatment and clinical metabolic control [8,17,19]. Other results do not provide evidence of the beneficial effect of a strict dietary regimen on the course of the LCHADD chorioretinopathy even if strict adherence to therapy was only observed in one patient [20].

Lowering 3-OHACs or 3-OHFAs could slow the progression of the retinopathy [8,17,19]. The link between 3-OHAC and 3-OHFA levels and diet is not fully clear. It has been postulated that the concentration of plasma 3-OHACs is related to the dietary fat intake of children with LCHADD [27], but these conclusions have been recently revisited [28]. Nevertheless, the role of diet, particularly the limitation of the lipid load, in preventing retinopathy is suggested by the results of studies conducted on patient-specific induced pluripotent stem cell-derived RPE cells. The authors observed that RPEs have a limited capacity for lipid storage and LCHAD activity is necessary for RPE lipid processing. Excessive free fatty acids can impair normal cell signaling, are prone to peroxidation, and can cause cellular dysfunction and apoptotic cell death [25].

No current treatments completely normalize plasma 3-OHACs and 3-OHFAs, unique intermediates seen in LCHADD that are potentially toxic to the RPE. Visual function and retinal structure may continue to decline with advancing age in patients with LCHADD despite the therapy [8,19,20]. In LCHADD mouse models, a significant accumulation of 3-OHACs in the RPE/sclera has been observed in 15-month-old LCADD mice, suggesting that age may contribute to the pathological progression [21]. 

Conclusions about this outcome are challenging because most studies are poor in details and might have potential biases in recording dietary intake. Among the studies analyzed, none demonstrated a direct correlation between the percentage of total fats or MCTs and the outcomes related to retinopathy. Instead, the findings were primarily linked to the normalization of acylcarnitines, which, although considered metabolic biomarkers, are not strongly correlated with dietary fat intake. Moreover, none of the studies reported data on energy intake compared to individual requirements, which could have implications for growth and metabolic control. 

It is possible that chronic fat modulation in the diet is less important than emergency treatments coping with catabolic states and metabolic decompensations. Decreasing the number of metabolic crisis has been shown to delay or slow the progression of the retinopathy, suggesting that these moments of stress accelerate the progression of the retinopathy [17]. The increase in the levels of unmetabolized fatty acids and toxic FAO intermediates and the occurrence of hypoketotic hypoglycemia during catabolic states could cause late complications and progressive ocular findings. 

The last outcome was the role of specific nutritional supplements. 

Children with LCHADD are treated with a low-fat diet and risk essential fatty acid and fat-soluble vitamin deficiency. Low plasma levels of essential fatty acids, especially DHA, have been discussed as a contributing factor to retinopathy because of their important role in retinal function [12,13]. Gillingham et al. [17] observed that visual acuity tended to improve with DHA supplementation and showed a partial correlation with plasma DHA concentrations. Interestingly, some patients with normal baseline plasma DHA levels also experienced improved visual acuity following supplementation. However, other studies have reported that most patients who developed retinopathy had been receiving DHA supplementation from an early age at doses comparable to those recommended for healthy individuals [6,8,16]. The role of other nutritional supplements, such as vitamin A and antioxidants, is less defined and more debated. Recent evidence from iPSC-derived LCHADD RPE cells suggests that damage induced by oxidative stress and lipid peroxidation can be rescued by potent antioxidants, like N-acetyl-L-cysteine [23]. No clinical studies investigated the potential role of antioxidants. 

The studies included in the review have several limitations. The main limits are the patient population size and the heterogeneity of the studies. All studies have a sample size < 50 patients and have been considered as being at a risk of bias in the study design and/or at risk of imprecision for the estimation of the analyzed outcome. In the same way, most studies are retrospective in design or prospective without a control group, and therefore, most studies were assigned to a low level of evidence. Only two studies were assigned a moderate level of evidence because of their prospective design with a historical control group [6,19].

Other limitations are the following: only four studies included both ocular and metabolic assessments as part of their follow-up; data about the dietary follow-up, such as assessments of nutritional status, growth, and adherence to the diet, are almost never reported; no studies considered other clinical contributors for retinopathy, such as prematurity [29]. 

The main strengths of this review are the inclusion of all studies reporting dietary outcomes related to the risk of retinopathy in LCHADD patients, the application of the PICOS model for study selection, and the GRADE system for the assessment of evidence. In addition, our review included patients from Finland, Germany, the USA, Sweden, and Austria, increasing the general applicability of the results.

Future studies, including cohort studies and randomized controlled trials, are needed to reach conclusive results. In particular, future research should encourage more robust studies, ideally involving the use of a 7-day diary reviewed by an expert dietitian in metabolic disorders, reporting the clear and precise calculation of dietary intakes. This underscores the need for studies with a specific focus and robust data-collection methods, such as 7-day food diaries.

In addition, new studies are needed to evaluate the long-term effects of standardized dietary protocols and of different dietary supplements (e.g., DHA, triheptanoin). Moreover, more studies are necessary to evaluate, more comprehensively, the comparison between ERG results with biochemical markers. 

## 5. Conclusions

Early presentation, acute symptoms, and sub-optimal chronic treatment control seem to contribute to the retinal pathology and poorer visual outcomes in LCHADD patients. Newborn screening for fatty acid b-oxidation defects has allowed for the implementation of dietary therapy at an early stage and may reduce critical decompensation episodes and early hypoglycemia with the potential amelioration of retinal outcomes. Nevertheless, an early LCHADD diagnosis and treatment can often only delay the onset of the retinopathy, highlighting that our current treatment strategies are not adequate and retina-specific treatments are needed.

More data assessing the optimal diet regimen are required. Further research to determine the mechanisms underlying the retinal damage in LCHADD is needed to guide the identification of novel treatments. 

## Figures and Tables

**Table 1:** Population features, dietary interventions, modality of follow-up, main results related to retinopathy, study design, study limitations and level of evidence (GRADE).

Reference	Patients (n., Timing of Diagnosis, Genotype)	Age at Follow-up, Period of Observation, and Country/Region	Dietary Interventions and Dietary Follow-Up	Ocular and Metabolic Assessment	Main Results	Study Design	Study Limitations	Level of Evidence (GRADE)
Immonen T et al. 2016 [6]	n. 16 (homozygous 1528G>C mutation); 3 patients were diagnosed post-mortem, none via NBS. Age at diagnosis: birth-5 months (mean 0.27 years). Survival rate: 62.5% Control group: n. 28 patients with the same mutation diagnosed during 1976–1996; 24 patients were diagnosed postmortem. Survival rate: 14.3%	Age: 1–11 years Survival rate at study time: 62.5% compared with 14.3% of control group. Period: 1997–2010 vs. 1976–1996 Country: Finland	1997–2010 cohort: low-fat and high-CHO diet, 5% of calories from LCT, including essential fatty acids, such as DHA, linoleic acid, and a-linolenic acid. Fat-soluble vitamins supplemented. MCT providing 15% to 20% of calories. Children received continuous overnight feeding. From adolescence, bedtime uncooked cornstarch to allow 9 h of fasting. Extra calories from glucose polymer or MCT before physical exercise. Follow-up: detailed dietary recall every 3 months during infancy, then one to three times per year. 1976–1996 cohort: some did not receive the diet therapy at all.	Ocular assessment: ophthalmologic evaluation once a year. Metabolic assessment: serum free fatty acids, plasma fatty acid profiles, serum total and free carnitine, blood acylcarnitine profiles, CK.	Retinopathy was detected in 9/11 patients (81%) who survived more than 6 months after diagnosis; 7/11 patients mild retinopathy, 1/11 moderate, 1/11 undefined. Despite dietary therapy, the acylcarnitine concentration ratio – (C16OH+C18:10H+C18OH)/C2 remained elevated (range 0.04–0.361, median 0.08, normal < 0.017) during the follow-up period.	Prospective and retrospective	Limited study population	Moderate
Fahnehjelm et al. 2016 [8]	n. 12 (n. 2 diagnosed via NBS and n. 10 at a median age of 5.5 months, range 1 week-13 months) Genotypes: n.a.	Age: 3–26 years at the time of last examination; median age at the first ocular examination was 15 months (range 5 weeks–6 years). Period: 1990–2012 Country: Sweden	Low fat intake and essential fatty acid supplements started at the time of diagnosis; DHA supplements in 11/12 patients. Eight patients have continuous overnight feeding. Oral glucose polymer or iv glucose infusion during febrile infections.	Ocular assessment: best corrected visual acuity—BCVA, stereopsis, color vision, ocular alignment, slit-lamp, ophthalmoscopy and retinoscopy, OCT, and ERG (with rode-cone amplitude and latency evaluation). Metabolic assessment: dicarboxylic acids and/or 3-OHACs.	More than 80% of patients developed pathological or subnormal retinal function; 2/12 blindness or moderate visual impairment; 11/12 retinal pigmentation (median age 3.9 years, 14 months–6 years); 7/12 retinal atrophy; 1/12 retinal fibrosis. OCT showed retinal thinning in 3/6 patients examined. ERG was performed on 11 patients (median age at first: 6 years, 0.7–16.5 years median age at the latest: 14 years, range 3–22 years). It was pathological in 5 patients, subnormal in 4 and was related to poor clinical metabolic control and severe neonatal symptoms. Retinopathy was more pronounced in patients with neonatal symptoms. Repeated ERG revealed reduced function with increasing age in 4 cases No correlation between ERG amplitude or latency with the first and only measurements of 3-OHAC levels in three patients who presented ERG deterioration over age.	Retrospective	Limited study population, no control group	Low
Karall et al. 2015 [16]	n. 14 (9/14, 64% diagnosis via NBS, 5/14 with clinical symptoms), all homozygous for 1528G>C mutation Age at diagnosis: median 15 days (range 1 day–20 months)	Age at the time of last examination: median 7.6 years (range 0.9–15.3 years) Period: until 2013 Median follow-up period: 7.8 years Country: Austria, Germany	Fat-defined diet (median total fat 29% of energy intake, range 15–40%); MCT 20–80% of fat (median 62%). LCFA median 38% of fat and 11.3% of energy intake (range 3–24%). DHA supplementation range 89–267 mg/day (mean 143.4 mg/day) 4 patients treated with C7.	Ocular assessment: n.a. Metabolic assessment: n.a.	n. 8/14 (57%) retinopathy; it was clinically relevant (severe impairment of vision) only in one patient. Mean age when retinopathy was firstly diagnosed: 51.6 months in patients born before introduction of LCHADD into NBS program and diagnosed clinically (mean age at diagnosis 10.3 months, range 3–23 months), 39 months in patients showing symptoms before NBS results were available (mean age at diagnosis 15 days), 53 months in asymptomatic patients with positive NBS results.	Retrospective	Limited study population, no control group	Low
Gillingham et al. 2005 [17]	n. 14 children with LCHADD Timing of diagnosis: n.a. Age at initial presentation range 0–3 years (from Gillingham et al. 2003) Genotype: 11/14 homozygous or heterozygous for 1528G>C mutation	Age: 1–12 years at enrolment Follow-up period: 2–5 years Country: USA	Low-fat diet (LCFAs 13 ± 7% of total energy) and DHA supplementation 65–130 mg/day; 12 patients were orally supplemented with MCT and carnitine before the beginning oral DHA.	Physical and biochemical evaluations. Ocular assessment: ERG (with rode-cone amplitude). and visual acuity based on visual evoked potential (VEP). Evaluations were performed at baseline and annually following the initiation of DHA supplementation.	Four patients developed severe retinopathy. Patients with sustained low plasma 3-OHACs maintained higher ERG amplitudes with time compared to patients with chronically high 3-OHACs. Strong negative correlation between 3-OHAC concentration and phototransduction as measured via ERG (*p* = 0.0038). Visual acuity appeared to increase with time based on DHA supplementation (*p* = 0.051), and there was a trend for a positive correlation with plasma DHA concentrations (*p* = 0.075, R2 = 0.31).	Prospective, open trial	Limited study population, no control group Decompensations: defined as periods when the families had contacted the hospital during infections but no acute blood tests confirmed.	Low
Fahnehjelm et al. 2008 [18]	n. 10 (diagnosis median age of 1 month (2 days–13 months). Mean age at the time of first examination: 14.5 months One patient was diagnosed in the neonatal period before any symptoms had developed because of a medical history in previous children in the family; n.8/10 patients homozygous 1528 G>C, n.2/10 the same mutation on one allele, the other mutation has not yet been identified	Age: n.5/10 had >10 years; n.4/10 had 5–10 years; n.1/10 had <5 years Country: Sweden	Low fat intake (approximately 20% of calories), mainly composed of MCT and essential fatty acid supplements started at the time of diagnosis (including DHA), 9/10 patients had continuous night feeds. CHO-rich supplement or iv glucose infusion during febrile infections.	Ocular assessment: BCVA, stereopsis, color vision, ocular alignment, slit-lamp, ophthalmoscopy and retinoscopy, OCT, ERG (with mixed rode-cone amplitude and rod and cone response). Metabolic assessment: dicarboxylic acids and acylcarnitines	n.4/10 retinal pigmentations at the first ocular examination (median age of 11 months, range 5 weeks–2.7 years). ERG was moderately to severely pathological in 7/10 (70%) children at latest follow-up Retinopathy was less pronounced in patients with early diagnosis.	Retrospective	Limited study population, no control group	Low
Gillingham MB et al. 2024 [19]	n. 28 LCHADD or TFP patients diagnosed via NBS (n. 20) or family history (n. 8) vs. n. 12 diagnosed symptomatically Genotype: homozygous for 1528G>C mutation 6 in NBS/family history group (+20 heterozygous), 4 in symptomatic group (+7 heterozygous)	Age at enrolment: NBS/family history 2–36 years; symptomatically diagnosed: 7–31 years Country: USA	Fasting avoidance, dietary long-chain fat restriction (100% patients), MCT (79% vs. 67%), C7 (50% vs. 33%), and/or carnitine supplementation (50% vs. 33%).	Ocular assessment: measures of visual acuity (Electronic Visual Acuity Tester with Logarithmic Minimal Angle of Resolution -LOGMAR), ERG (with rode-cone amplitude and latency), fundal imaging, contrast sensitivity, and visual fields tests. Metabolic assessment: n.a.	Visual acuity, rod- and cone-driven amplitudes on ERG, contrast sensitivity scores, and visual fields were all significantly worse among participants diagnosed symptomatically compared to NBS (92% vs. 68%). In mixed-effects models, both age and presentation (symptomatic vs. NBS) were significant independent factors associated with visual outcomes. Older participants exhibited the most severe retinal changes.	Prospective	Some patients mainly had laboratory tests taken when they presented with clinical symptoms, and others were tested more frequently during regular scheduled outpatient clinic visits. This means that the laboratory results were less reliable for evaluating the long-term overall metabolic control	Moderate
Dulz et al. 2021 [20]	n. 6 (3 diagnosed via NBS, age 8–14,8 years; n.1 diagnosed within 1 y of life; n. 2 diagnosed later with symptoms). Genotype: 5 homozygous for 1528G>C mutation, one patient with not further identified compound heterozygous mutation	Age: mean 14.9 years, range 7.9–23.9 years Mean follow-up period: 6.2 years (2.8–9.6 years) Country: Germany	A permanent low-fat diet, including the supplementation of essential fatty acids, was initiated at the time of diagnosis and strict adherence to therapy was only observed in 1 patient.	Ocular assessment: visual acuity (BCVA), fundus imaging, OCT, fundus autofluorescence, and infrared imaging Metabolic assessment: n.a.	All patients showed retinal alterations, but early diagnosis was associated with a milder phenotype and a longer preservation of visual function. Among symptomatic patients, only one showed mild retinal involvement at the time of diagnosis. No obvious correlation was present among 3-OHFA levels, the number of metabolic crisis, and the stage and ocular progression. Early diagnosis does not prevent retinopathy but might contribute to a milder phenotype	Retrospective	Very limited study population	Low

## Data Availability

All databases generated for this study are included in the article.

## Abbreviations

DHA	docosahexaenoic acid
ERG	electroretinogram
FAO	fatty acid β-oxidation
LCFA	long-chain fatty acids
LCHAD	long-chain 3-hydroxyacyl-CoA dehydrogenase
LCHADD	long-chain 3-hydroxyacyl-CoA dehydrogenase deficiency
LCT	long-chain triglycerides
MCT	medium-chain triglyceride
NBS	newborn screening
OCT	optical coherence tomography
RPE	retinal pigment epithelial cells
TFP	trifunctional protein
TFPD	TFP deficiency
3-OHACs	3-hydroxyacylcarnitines
3-OHFAs	3-hydroxy fatty acids

## References

[1] Prasun P., LoPiccolo M.K., Ginevic I., Adam M.P., Feldman J., Mirzaa G.M., Pagon R.A., Wallace S.E., Bean L.J.H., Gripp K.W., Amemiya A. (1993–2024). Long-Chain Hydroxyacyl-CoA Dehydrogenase Deficiency/Trifunctional Protein Deficiency. GeneReviews(R) [Internet].

[2] Vockley J. (2020). Long-chain fatty acid oxidation disorders and current management strategies. Am. J. Manag. Care.

[3] Spiekerkoetter U., Bastin J., Gillingham M., Morris A., Wijburg F., Wilcken B. (2010). Current issues regarding treatment of mitochondrial fatty acid oxidation disorders. J. Inherit. Metab. Dis..

[4] Spiekerkoetter U., Lindner M., Santer R., Grotzke M., Baumgartner M.R., Boehles H., Das A., Haase C., Hennermann J.B., Karall D. (2009). Treatment recommendations in long-chain fatty acid oxidation defects: Consensus from a workshop. J. Inherit. Metab. Dis..

[5] Gillingham M., Van Calcar S., Ney D., Wolff J., Harding C. (1999). Dietary management of long-chain 3-hydroxyacyl-CoA dehydrogenase deficiency (LCHADD). A case report and survey. J. Inherit. Metab. Dis..

[6] Immonen T., Turanlahti M., Paganus A., Keskinen P., Tyni T., Lapatto R. (2016). Earlier diagnosis and strict diets improve the survival rate and clinical course of long-chain 3-hydroxyacyl-CoA dehydrogenase deficiency. Acta Paediatr..

[7] Fletcher A.L., Pennesi M.E., Harding C.O., Weleber R.G., Gillingham M.B. (2012). Observations regarding retinopathy in mitochondrial trifunctional protein deficiencies. Mol. Genet. Metab..

[8] Fahnehjelm K.T., Liu Y., Olsson D., Amrén U., Haglind C.B., Holmström G., Halldin M., Andreasson S., Nordenström A. (2016). Most patients with long-chain 3-hydroxyacyl-CoA dehydrogenase deficiency develop pathological or subnormal retinal function. Acta Paediatr..

[9] Tyni T., Immonen T., Lindahl P., Majander A., Kivela T. (2012). Refined staging for chorioretinopathy in long-chain 3-hydroxyacyl coenzyme A dehydrogenase deficiency. Ophthalmic Res..

[10] Wongchaisuwat N., Gillingham M.B., Yang P., Everett L., Gregor A., Harding C.O., Sahel J.A., Nischal K.K., Scanga H.L., Black D. (2024). A proposal for an updated staging system for LCHADD retinopathy. Ophthalmic Genet..

[11] Harding C.O., Gillingham M.B., van Calcar S.C., Wolff J.A., Verhoeve J.N., Mills M.D. (1999). Docosahexaenoic acid and retinal function in children with long-chain 3-hydroxyacyl-CoA dehydrogenase deficiency. J. Inherit. Metabol. Dis..

[12] Swinkels D., Baes M. (2023). The essential role of docosahexaenoic acid and its derivatives for retinal integrity. Pharmacol. Ther..

[13] Shimazawa M., Nakajima Y., Mashima Y., Hara H. (2009). Docosahexaenoic acid (DHA) has neuroprotective effects against oxidative stress in retinal ganglion cells. Brain Res..

[14] Page M.J., McKenzie J.E., Bossuyt P.M., Boutron I., Hoffmann T.C., Mulrow C.D., Shamseer L., Tetzlaff J.M., Akl E.A., Brennan S.E. (2021). *The PRISMA* 2020 statement: An updated guideline for reporting systematic reviews. BMJ.

[15] Schünemann H.J., Brennan S., Akl E.A., Hultcrantz M., Alonso-Coello P., Xia J., Davoli M., Rojas M.X., Meerpohl J.J., Flottorp S. (2023). The development methods of official GRADE articles and requirements for claiming the use of GRADE—A statement by the GRADE guidance group. J. Clin. Epidemiol..

[16] Karall D., Brunner-Krainz M., Kogelnig K., Konstantopoulou V., Maier E.M., Möslinger D., Plecko B., Sperl W., Volkmar B., Scholl-Bürgi S. (2015). Clinical outcome, biochemical and therapeutic follow-up in 14 Austrian patients with Long-Chain 3-Hydroxy Acyl CoA Dehydrogenase Deficiency (LCHADD). Orphanet J. Rare Dis..

[17] Gillingham M.B., Weleber R.G., Neuringer M., Connor W.E., Mills M., van Calcar S., Ver Hoeve J., Wolff J., Harding C.O. (2005). Effect of optimal dietary therapy upon visual function in children with longchain 3-hydroxyacyl CoA dehydrogenase and trifunctional protein deficiency. Mol. Genet. Metab..

[18] Fahnehjelm K.T., Holmstrom G., Ying L., Haglind C.B., Nordenstrom A., Halldin M., Alm J., Nemeth A., Von Döbeln U. (2008). Ocular characteristics in 10 children with long-chain 3-hydroxyacyl-CoA dehydrogenase deficiency: A cross-sectional study with long-term follow-up. Acta Ophthalmol..

[19] Gillingham M.B., Choi D., Gregor A., Wongchaisuwat N., Black D., Scanga H.L., Nischal K.K., Sahel J.A., Arnold G., Vockley J. (2024). Early diagnosis and treatment by newborn screening (NBS) or family history is associated with improved visual outcomes for long-chain 3-hydroxyacylCoA dehydrogenase deficiency (LCHADD) chorioretinopathy. J. Inherit. Metab. Dis..

[20] Dulz S., Atiskova Y., Engel P., Wildner J., Tsiakas K., Santer R. (2021). Retained visual function in a subset of patients with long-chain 3-hydroxyacyl-CoA dehydrogenase deficiency (LCHADD). Ophthalmic Genet..

[21] Babcock S.J., Curtis A.G., Gaston G., Elizondo G., Gillingham M.B., Ryals R.C. (2024). The LCHADD Mouse Model Recapitulates Early-Stage Chorioretinopathy in LCHADD Patients. Investig. Ophthalmol. Vis. Sci..

[22] Gaston G., Babcock S., Ryals R., Elizondo G., DeVine T., Wafai D., Packwood W., Holden S., Raber J., Lindner J.R. (2023). A G1528C Hadha knock-in mouse model recapitulates aspects of human clinical phenotypes for long-chain 3-hydroxyacyl-CoA dehydrogenase deficiency. Commun. Biol..

[23] DeVine T., Elizondo G., Gaston G., Babcock S.J., Matern D., Shchepinov M.S., Pennesi M.E., Harding C.O., Gillingham M.B. (2024). iPSC-Derived LCHADD Retinal Pigment Epithelial Cells Are Susceptible to Lipid Peroxidation and Rescued by Transfection of a Wildtype AAV-HADHA Vector. Investig. Ophthalmol. Vis. Sci..

[24] Tucci S. (2022). An Altered Sphingolipid Profile as a Risk Factor for Progressive Neurodegeneration in Long-Chain 3-Hydroxyacyl-CoA Deficiency (LCHADD). Int. J. Mol. Sci..

[25] Polinati P.P., Ilmarinen T., Trokovic R., Hyotylainen T., Otonkoski T., Suomalainen A., Skottman H., Tyni T. (2015). Patient-specific induced pluripotent stem cell-derived RPE cells: Understanding the pathogenesis of retinopathy in long-chain 3-hydroxyacyl-CoA dehydrogenase deficiency. Investig. Ophthalmol. Vis. Sci..

[26] Kauppinen A., Paterno J.J., Blasiak J., Salminen A. (2016). Kaarniranta. Inflammation and its role in age-related macular degeneration. Cell. Mol. Life Sci..

[27] Gillingham M.B., Connor W.E., Matern D., Rinaldo P., Burlingame T., Meeuws K., Harding C.O. (2003). Optimal dietary therapy of long-chain 3-hydroxyacyl-CoA dehydrogenase (LCHAD) deficiency. Mol. Genet. Metab..

[28] Gillingham M.B., Choi D., Gregor A., Pennesi M., Nischal K., Sahal J.A., Vockley J., Matern D., Harding C. (2024). Metabolomics, acylcarnitines, genotype, visual acuity and diet among 39 patients with LCHADD/TFPD. Abstracts. J. Inherit. Metab. Dis..

[29] Sabri K., Ells A.L., Lee E.Y., Dutta S., Vinekar A. (2022). Retinopathy of Prematurity: A Global Perspective and Recent Developments. Pediatrics.

